# Contributions to Operative Surgery and Surgical Pathology

**Published:** 1858-07

**Authors:** 


ART. V.—*Contributions to Operative Surgery and Surgical Pathology.* By J. M. CARNOCHAN, M.D., Professor of Surgery in the New York Medical College, Surgeon-in-chief to the State Emigrant's Hospital, etc. With illustrations drawn from nature. Philadelphia: Lindsay & Blakiston, 1858, 4to. pp. 32.

THE present number of this work, the first of a series of twelve, comprises an account of amputation of the entire lower jaw, with general remarks on this operation, and several cases of elephantiasis Arabum of the inferior extremity, treated by ligature of the femoral artery. The articles are illustrated by excellent lithographic drawings, and are treated with circumstantial minuteness, having been previously published in the New York Journal of Medicine. The mechanical execution is altogether superior, and reflects great credit upon the publishers, Messrs. Lindsay and Blakiston. The work, when complete, will form a beautiful volume of at least five hundred pages, and be the most valuable original contribution to the art and science of surgery that has yet been made by an American surgeon.

To enable the reader to obtain a correct idea of the various topics that will be discussed in the succeeding numbers, we subjoin the table of contents of each:—

"No. 2. Case of exsection of the entire ulna. Remarks on neuralgia, with three cases successfully treated by exsection of the second branch of the fifth pair of nerves, beyond the ganglion of Meckel.

"No. 3. Case of restoration of the entire upper lip. Remarks on the pathology of congenital dislocations of the hip-joint, with illustrations.

"No. 4. Case of exsection of the entire radius. Case of exsection of the three lower fourths of the same bone. Remarks on osteo-aneurism, with a case.

"No. 5. Case of amputation at the shoulder-joint for the removal of a large osteo-fibro-cancerous tumor of the humerus, with remarks on amputation at this joint. Case of penetrating gun-shot wound of the heart.

"No. 6. Case of double congenital dislocation of the hip-joint. Remarks on double capital operations, with cases. Remarks on the comparative merits of the partial amputations of the foot. Remarks on amputations through the ankle-joint.

"No. 7. Successful removal of a large fibro-cartilaginous tumor, growing from the sixth and seventh ribs, over the region of the heart. Remarks on the treatment of varicose veins of the lower extremities, with cases. Remarks on the creation of an artificial joint upon the lower jaw, in a case of complete anchylosis at the temporo-maxillary articulation of one side.

"No. 8. Remarks on the operation of double complicated hare-lip, with cases. Remarks on the etiology of congenital dislocations of the hip-joint. Remarks on the removal of the first dressings after capital amputations.

"No. 9. Case of encysted sanguineous tumor of the neck successfully removed, with remarks on such formations. Remarks on the purulent ophthalmia of large and crowded institutions. Case of vesico-vaginal fistula and stricture of the vagina, with formation of two large urinary calculi in the vagina, behind the stricture; spontaneous cure of the fistula.

"No. 10. Two cases of amputation at the hip-joint. Remarks on the anatomy of femoral hernia. Case of epilepsy treated by tracheotomy, and wearing of a tracheal tube, with remarks. Remarks on the restoration of the entire lower lip, with cases. Cases of amaurosis treated with the Pommade de Gondret on the sinciput."

Dr. Carnochan has enjoyed rare opportunities of observing surgical diseases at the Emigrant's Hospital, of which he has now been chief surgeon for upwards of eight years, and his ability as a brilliant and original operator is well known to the profession of this country and of Europe. We bespeak for the work an attentive perusal.

